# Engineering CaO@activated carbon nanocomposite for simultaneous energy storage and pollutant adsorption

**DOI:** 10.1039/d6ra01528j

**Published:** 2026-04-28

**Authors:** Arsh E Noor, Shafaqat Ali, Awais Ahmad

**Affiliations:** a Department of Environmental Sciences, Government College University Faisalabad Pakistan shafaqataligill@gcuf.edu.pk; b Department of Chemistry, The University of Lahore Lahore 54590 Pakistan awaisahmed@gcuf.edu.pk

## Abstract

A nanoconfinement approach was employed in this study to encapsulate activated carbon with calcium oxide NPs. Here, we present an innovation in materials science by introducing a CaO@AC bifunctional catalyst with unique crystallographic structure and outstanding properties for wastewater treatment and energy storage. This catalyst enables the fast degradation of cocktail pollutants within a minimum time and is useful for the storage of clean energy. The photocatalytic degradation of rhodamine-B, rose bengal, and methylene blue was successfully performed by the bifunctional catalyst with 95%, 74%, 86% degradation efficiencies, respectively, within 40 min. The bifunctional catalyst showed a low charge transfer resistance, decreasing from 1.81 Ω before charge–discharge cycling to 1.62 Ω after cycling, indicating faster ionic diffusion, higher structural stability, and increased surface activation of the electrode. This catalyst achieved a high specific capacity of 230 F g^−1^ at 4 A g^−1^ in 3 M KOH and retained approximately 99% of its initial capacitance after 5000 cycles. Additionally, the doped CaO@AC nanocomposite exhibited an enlarged CV area compared with the AC electrode, indicating an enhanced electron adsorption capacity due to the surface functionalities of the CaO@AC matrix. This work paves the way for the further exploration of the innovative CaO@AC bifunctional catalyst as a promising candidate for sustainable development.

## Abstract 2

A nanoconfinement approach was employed in this study to encapsulate activated carbon with calcium oxide NPs.

## Graphical abstract

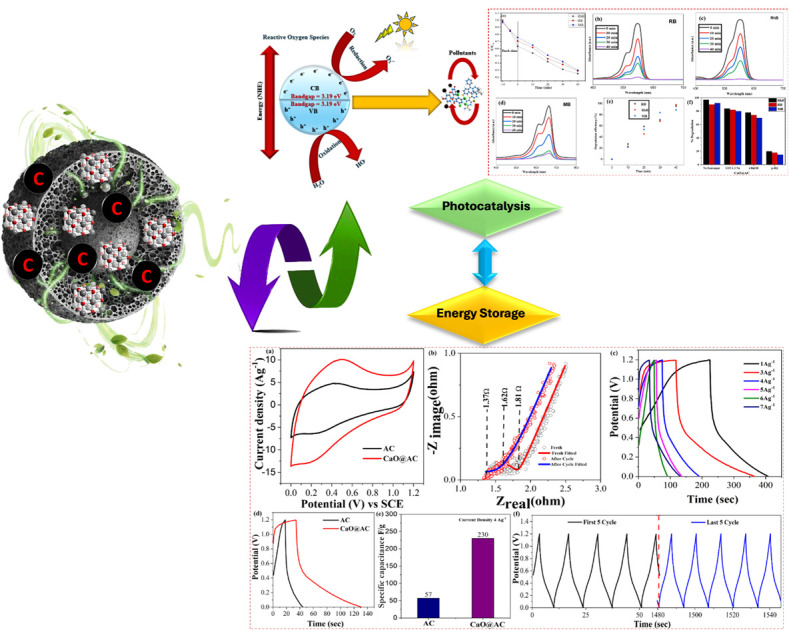

## 1. Introduction

The rising demand for energy and water is putting stress on the environment, eventually resulting in environmental degradation and resource depletion.^1^ The industrial revolution led to the emergence of energy crises and water pollution. The textile industry is a major contributor to water pollution and releases huge amounts of pollutants directly into water bodies, deteriorating the quality of the water.^2^ These emerging pollutants produce antibiotic-resistant bacteria (ARG), and hence, there is an urgent need for the remediation of such pollutants.^3–5^ The presence of dyes in water bodies can cause negative impacts on aquatic systems and living organisms^6^ by inducing mutagenic and carcinogenic properties,^7^ reducing photosynthetic activities^8^ and blocking the penetration of light to microbes.^9^ In general, the dyes can be separated in multiple catagories but non-ionic dyes (dispersion dyes, vat dyes),^10^ cationic dyes,^11^ and anionic dyes (direct dyes, acid dyes, and reactive dyes)^12^ are known as major catagories of dyes. Cationic dyes have a hazardous impact on water bodies and the overall environment. Cationic dyes can cause kidney failure, developmental retardation, and autoimmune disorders in human beings and other organisms, which ultimately lead to death.^13,14^

The increasing demand for fossil fuels ultimately leads to unprecedented pollution levels in urban and rural areas, which increases the rate of global pollution.^15^ There has been a drastic increase in the use of sustainable energy resources owing to the increasing trend of depleting natural reserves. There has been a renewed interest in electric vehicles as an alternative to combustion engine vehicles, which are responsible for 25% of greenhouse gas emissions.^16^ The urgent need to obtain long-term energy storage has triggered the active study of new storage methods and thus made energy equipment one of the focal points of modern scientific study.^17^ Supercapacitors are an interesting type of energy storage device, characterised by high cyclability and excellent power density capabilities. In particular, electrochemical double-layer capacitors (EDLCs) have gained interest due to their high power density, fast charge–discharge response and long cycle life, making them one of the most viable solutions for energy storage.^18^ However, the commercially available EDLCs exhibit an energy density less than 10 Wh kg^−1^, which is a serious drawback that prevents its widespread application.^19^ An important factor in determining the performance of supercapacitors is the electrode materials. Therefore, improvements in the electrochemical capacity of EDLCs necessitate designing materials that exhibit efficient charge storage capacity. It has recently been highlighted in the literature that macro-, meso-, and micro-porous carbon nanomaterials are potential electrode materials that can attain better performance metrics. Particularly, microporous carbon nanomaterials provide a large surface area for the adsorption of ions, which increases capacitance and energy density.^20^ Macropores act as ion-buffer reservoirs, reducing diffusion barriers to deeper pore networks, and mesopores offer specific sites for charge storage and increase ion transport.^21^ As a result, out of multiple types of porous materials, which include zeolites and porous silica, such structures are one of the most desirable porous materials to be used as an electrode in EDLCs.^22^

Nanotechnology is an emerging technology for developing wastewater treatment and addressing the energy crisis. Different methods including coagulation, reverse osmosis,^23^ photodegradation,^8^ chemical oxidation,^24^ membrane filtration,^25^ adsorption treatment,^26^ chemical precipitation,^27^ solvent extraction, nanofiltration, electrochemical performance, ozonation, and biosorption are used for wastewater treatment.^28^ Some of these methods suffer from disadvantages such as high costs, minor sludge formation, and higher electrical power and chemical reagent requirements. In this regard, nanotechnology opens a new direction to overcome such challenges. With help of photocatalytic degradation no sludge created and tainted aromas are much reduced, but they can effortlessly form by-products.^29,30^ Photodegradation is one of the most well-known approaches for dye degradation due to its high efficiency, non-toxicity, low cost, and easy operation. Practical treatment techniques are required for the efficient treatment of cationic contaminants in wastewater, which is a critical issue. Many biosorbents, such as metal oxides, zeolites, multiwalled carbon nanotubes, and activated carbon derived from cellulose, are employed in dye treatment processes and are successfully used in wastewater remediation.^31,32^ Notable metal oxides (MOs) that are widely used in photocatalytic processes include TiO_2_, ZnO, Fe_2_O_3_, SnO_2_, VO_*x*_, CuO, CaO, Co_3_O_4_, and MoO_*x*_.^33,34^ In particular, CaO has gained considerable attention as it is an inexpensive, non-corrosive, environmentally friendly, and recyclable material.^35,36^

CaO properties includes an effective additive, dopant, drug delivery agent, chemotherapeutic agent, gas purifier, catalyst in LEDs, hazardous chemical removal helper, and gas purification partner. Due to its many uses, calcium oxide (CaO) is a highly valued and sustainable substance that can be used to solve various environmental and industrial problems. Three crucial factors need to be carefully considered to ensure the high yield and purity of the synthesized metal oxides: (i) solvent selection, (ii) reducing agent use, and (iii) capping agent use (non-hazardous substance). Eliminating environmental toxicity is a major goal for researchers, especially regarding waterborne and bacterial illnesses that may be managed using nanoparticles. One of the most often utilized adsorbents is activated carbon (AC), which is well known for its porous structure, large specific surface area, and moderate adsorption capability. Interestingly, AC is a powerful catalytic support and has a high efficiency for pollutant adsorption. In the context of energy storage, carbon-blended metal oxides are used as electrode materials for energy storage devices such as TiO_2_/AC,^37^ CuO/AC, ZnO/AC, V_2_O_5_/AC, and Ce–Zr/AC. This study incorporates AC into CaO by following a straightforward green synthesis method. *Manilkara zapota*, a member of the Sapotaceae family, is recognized by various names such as sapodilla tree, naseberry tree, and nispero tree. Various components in this plant are used for the treatment of tenderness, pain, fever, coughs, diarrhea, and dysentery. Myricetin-3-o-α-l-rhamnoside, 3β-hydroxyolean-12-en-28-oic acid, lupeol acetate, 3,4-dihydroxy-trans-cinnamate, β-d-fructofuranosyl α-d-glucopyranoside, and (9*Z*)-octadec-9-enoic acid are some of the significant components found in the *Manilkara zapota* leaves.

The chemicals that were extracted from the leaves have antibacterial properties. Herein, CaO blended with AC NPs (CaO@AC) was synthesized by utilizing the extract of *M. zapota* plants as a reducing agent. Activated carbon provides numerous active sites for the adsorption of organic contaminants, with its surface serving as the adsorption substrate. The adsorbed molecules are then transferred to the surface of the photoactive CaO, which speeds up the breakdown process of contaminants. Different analyses such as FTIR spectroscopy, XRD, TEM, SEM, and EDX were performed to characterize the nature of the synthesized photocatalysts. By employing a UV-vis spectrophotometer, the nanoparticle's photocatalytic activity was evaluated. The electrochemical properties of CaO@AC were analyzed using CV, GCD, and EIS analyses. The novelty and importance of the CaO@AC catalyst lies in the synergistic combination of CaO nanoparticles with activated carbon (AC) that offers superior catalytic properties over other reported AC-supported systems of metal oxides. Contrary to numerous commonly used metal oxide/AC catalysts, which largely depend on the dispersion of the transition metal oxides, the CaO-based systems have strong basic active sites that are provided by CaO along with the advantages of the high surface area, porosity, and electron-transfer capacity of the activated carbon. This combination leads to the enhanced adsorption of reactants and transference of charges and increased accessibility of catalytic sites. The CaO@AC material has benefits over the state-of-the-art AC-supported catalysts in terms of cost and environmental friendliness because it utilizes an inexpensive and non-toxic alkaline earth oxide and offers excellent catalytic stability. Moreover, the homogeneous surface distribution of CaO on the AC surface aids in preventing the aggregation of NPs, thus increasing the active sites and response to catalysis of the overall process. This distinguishes the current work from the literature and illustrates the prospects of CaO@AC as a promising and sustainable catalyst to develop further catalytic applications.

## 2. Experimental

### 2.1. Preparation of *Manilkara zapota* leaf extract

Approximately 500 mg of *Manilkara zapota* leaves were washed with distilled water and dried at room temperature for 15 to 20 minutes. A 500 mL beaker containing 200 mL of distilled water and dried leaves was heated for an hour at 100 °C to obtain the extract solution. The CaO@AC nanocomposite was synthesized using freshly produced *Manilkara zapota* leaf extract.

### 2.2. Synthesis of the CaO@AC nanocomposites

**Fig. 1:**
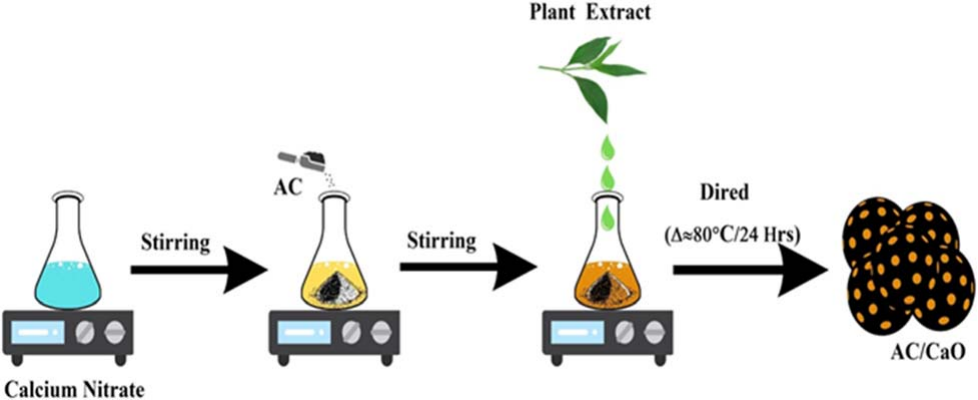
Schematic of the synthesis of CaO@AC (temperature: 80 °C; time: 24 h).

In a 250 mL RB flask, about 90 mL of 1 M calcium nitrate, 2 g of activated carbon, 10 mL of fresh leaf extract, and 20 mL of distilled water were added. The mixture was heated at 60 °C and stirring constantly for six hours. Using the fresh leaf extract of *Manilkara zapota*, the ions of calcium nitrates were transformed to CaO NPs. The colour of the solution changed from yellow to yellowish brown, which is a clear sign of the formation of CaO NPs. Ultimately, DM was used to neutralize this solution and repeatedly cleaned. Next, the precipitate was separated using a centrifuge running for three minutes at 10 000 rpm. Finally, the precipitate was washed and dried in a hot air oven for 24 hours at 80 °C. Fig. 1 shows the synthesis of CaO@AC following a green-mediated approach.

### 2.3. Characterization

X-ray diffraction (XRD) were analyzed using PANalytical B.V. (Netherlands). By a powder sample approach, the Fourier transform infrared (FTIR) spectra of the nanocomposite were recorded using a PerkinElmer, within the range of 400 to 4000 cm^−1^. Using scanning electron microscopy (SEM) and EDX at an accelerating voltage of 30 kV, the surface morphology of the nanoparticle sample was investigated (Carl Zeiss). FEI Titan 80–300 transmission electron microscope (TEM) was used with a 200 kV acceleration voltage. Physical Electronics performed an X-ray photoelectron spectroscopy (XPS) experiment. PHI 5000 Versa Probe III is the model number. BaSO_4_ was utilized as the reflectance standard while recording the ultraviolet-visible (UV-vis) diffuse reflectance spectra of the material using a spectrophotometer (UV-2700, Shimadzu).

### 2.4. Photocatalytic performance evaluation

Sunlight simulators were used to catalyze the photocatalytic degradation of RhB, RB, and MB dyes. A 250 mL beaker was filled with 50 mL of RhB (1 × 10^−5^ mol) to initiate the reaction. The mixture was mixed with approximately 0.05 grams of photocatalyst and left in the dark for two hours to allow for adsorption and desorption kinetics. The mixture was continuously stirred at 550 rpm for two hours while exposed to simulated sunlight irradiation. The solution was taken out at 10 minutes intervals and centrifuged. The rate of dye degradation was computed using a spectrophotometer by measuring the decrease in absorbance at its characteristic maximum wavelength (*λ*_max_ = 554). The following formula was utilized to determine the percentage of degradation kinetics of the dye:^38^1
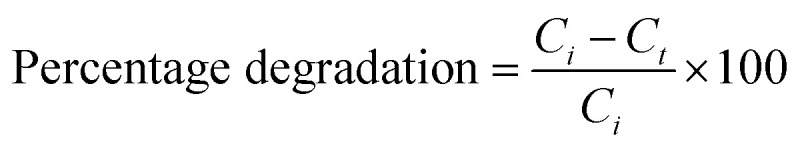
where: *C*_*i*_ = initial dye concentrations. *C*_*t*_ = dye concentrations at time *t*.

### 2.5. Antimicrobial activities

*Staphylococcus aureus* 9779 and *Escherichia coli* 745 were used for the antibacterial test. *E. coli* and *S. aureus* were cultured in an LB medium for 48 hours at 36 °C. The Luria–Bertani medium could cultivate Gram-positive and Gram-negative bacteria. Then, diffusion method was used to examine the created nanocomposite and the reference sample. Wells were prepared using a cork borer (0.85 cm). Each well was filled with around 50 µL of the test chemical, which was then incubated at 36 °C overnight. The microorganism growth was assessed by measuring the diameter of the zone of inhibition. Zone diameter measurements were collected, and the experiment was run five times to identify the mean values. The outcomes were contrasted with those of the antibacterial *Streptomycin* disc (20 mm) standard.

### 2.6. Electrochemical measurements

A three-electrode system was fabricated to determine the electrochemical properties of the CaO@AC electrode. The working electrode was prepared as follows: 80 wt percent of the active material that was synthesized, 15% wt of AC, and 5% wt An appropriate amount of polyvinylidene difluoride (PVDF) was mixed with 1.0 mL of *N*-methyl-2-pyrrolidone (NMP) and stirred in an ultrasonic bath. The slurry was then spurted on a 1 cm^2^ graphite substrate paper, and it was dried in an oven at a high temperature for several hours. The active material deposited was about 1.5 mg cm^−2^. In this work, the calomel electrode was a saturated cell with a Pt reference and a counter electrode.^39^ The electrochemical properties of the active material were examined using cyclic voltammetry (CV), galvanostatic charge/discharge (GCD), and electrochemical impedance spectroscopy (EIS). The capacitance parameters including specific capacitance were calculated using eqn (2) from the GCD traces:2
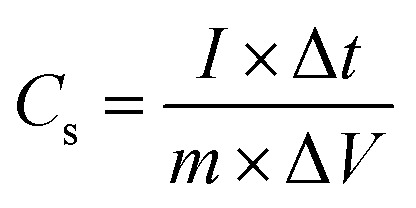
In the mentioned formulas, *I*, Δ*t*, *m*, and Δ*V* are the current intensity (A), time of discharge (s), amount of active material on the electrode surface (g), potential range of charge/discharge (V), and specific capacitance (F g^−1^), respectively.^40^

3 *C* = (*i* × *t*)/(*m* × *V*)

4 *E* = (1/2) *CV*^2^ = 0.1388*CV*^2^

5 *P* = (*E*/*t*) × 3600

The electrochemical performance of the electrode was evaluated by calculating the specific capacitance, energy density, and power density using standard equations. The specific capacitance (*C*) was determined from the galvanostatic charge–discharge (GCD) curves using the relation 
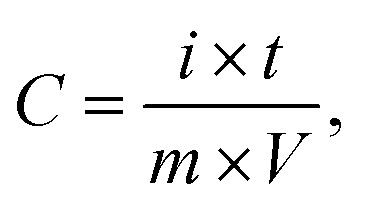
 where *i* is the discharge current, *t* is the discharge time, *m* is the mass of the active material, and *V* is the potential window. The energy density (*E*) was then calculated using 
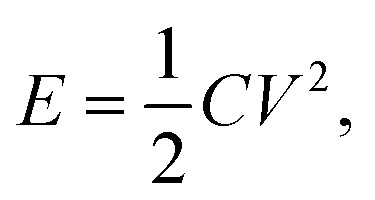
 which can also be expressed as *E* = 0.1388*CV*^2^ when converted into practical units (Wh kg^−1^). Furthermore, the power density (*P*) was obtained using the relation 
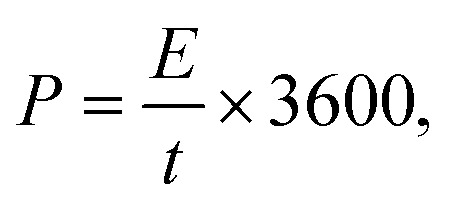
 where *t* is the discharge time in seconds. These equations provide a comprehensive evaluation of the charge storage capability, energy delivery, and rate performance of the electrode material, enabling a direct comparison with previously reported supercapacitor systems.

## 3. Results and discussions

### 3.1. Physicochemical analysis of CaO@AC

**Fig. 2:**
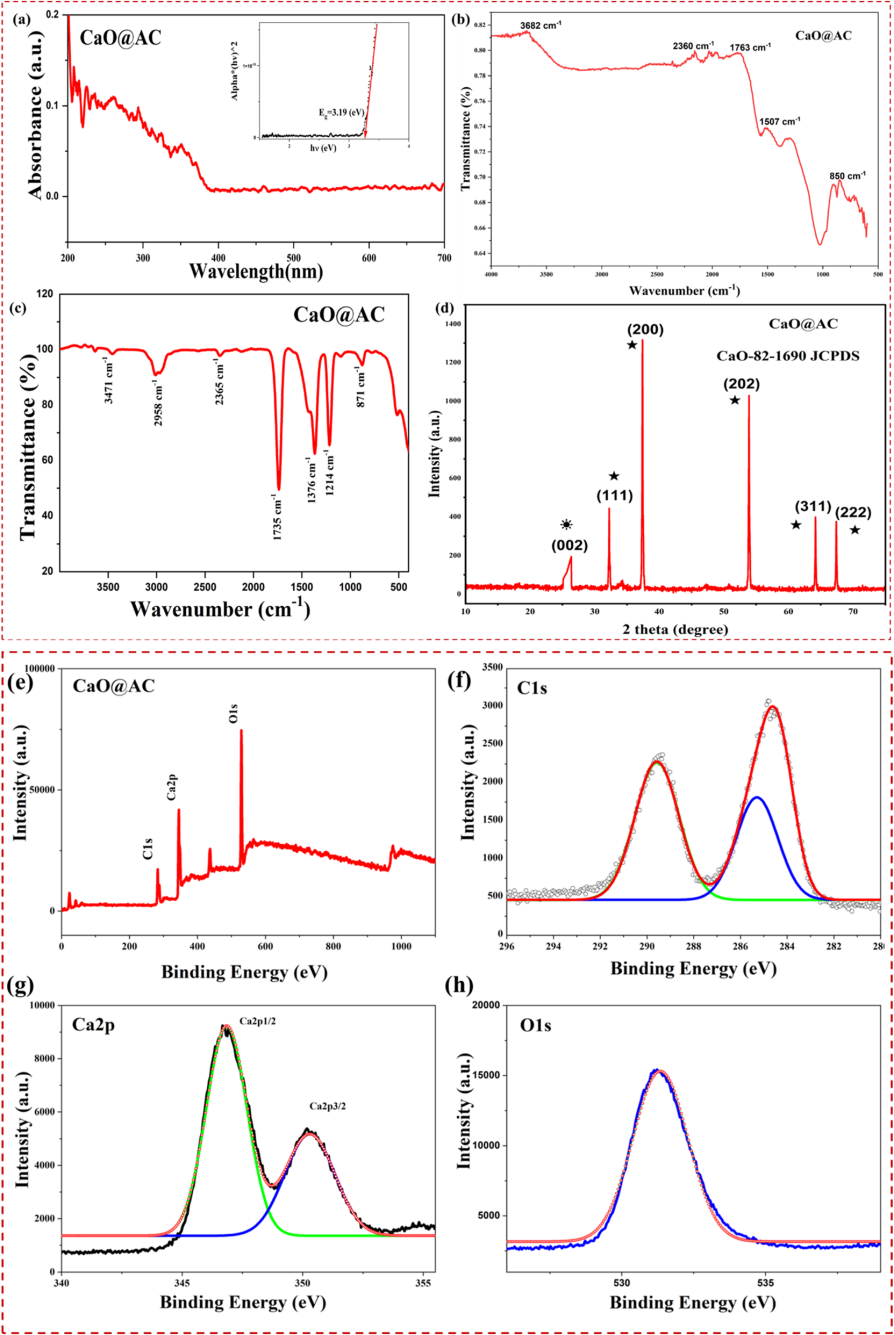
(a): UV-DRS spectra of the CaO@AC nanocomposite, with the inset featuring its Tauc plot. Experimental conditions: DRS analysis was performed using a Shimadzu UV-2700 spectrophotometer with BaSO_4_ as the reflectance standard. (b and c) FTIR spectra of the CaO@AC nanocomposite. Experimental conditions: FTIR measurements were taken using a PerkinElmer spectrometer in the range of 400–4000 cm^−1^. (d) XRD spectra of the CaO@AC nanocomposite. Experimental conditions: XRD analysis was performed using PANalytical B.V., Netherlands, with a Cu-Kα radiation source. High-resolution XPS (e) survey spectra and (f) C 1s, (g) Ca 2p, and (h) O 1s spectra of the CaO@AC nanocomposite. Experimental conditions: XPS analysis was conducted using a PHI 5000 Versa Probe III with a monochromatic Al-Kα source.

Fig. 2(a) presents the diffuse reflectance spectroscopy (DRS) results of CaO@AC. From the spectra, it is clear that only high absorption wavelengths in the UV (*λ* < 290–350 nm) are shown by the CaO@AC nanocomposite. However, the CaO@AC nanocomposite exhibits absorption bands in the visible and near-infrared frequencies. This distinct behavior, which reflects the improved semiconductor performance of the as-prepared CaO@AC nanocomposite, is caused by CaO doping in the AC matrix. Additionally, from the Tauc plot the CaO@AC nanocomposite, its band gap was 3.19 eV (Fig. 2(a)). The band gap is obtained using eqn (3). This finding points to the large bandgap present in the nanoparticle material. These results show that the increased absorptions of visible light facilitated by CaO resulted in the much higher photocatalytic activity of the CaO@AC nanocomposite.

6 (*αh*_*v*_)*n* = *A*(*h*_*v*_ − *E*_g_)

where *α* is the absorption coefficient, *h*_*ν*_ is the photon energy, *A* is a constant, and *n* denotes the type of electronic transition.

The infrared spectrum given in Fig. 2(b) for CaO@AC gives detailed information about its structural composition. In Fig. 2(c), an O–H stretching vibration, characteristic of the OH group, on the plane of the AC is found as a sharp peak at about 3470 cm^−1^. Moreover, clear peaks, at 1735 cm^−1^ and 1376 cm^−1^, reveal the stretching and asymmetric bending vibrations corresponding to the C–H and C=O bonds, respectively, within the carboxyl moieties.^41^ These specific parts include aldehydes and keto groups embedded cyclically in the AC template. A characteristic feature is the broad peak at 871 cm^−1^, which is indicative of Ca–O.^42,43^ This bond stretching has been observed inside the doped CaO@AC nanocomposite. The highest intensity of metal–oxygen is the same as that reported in the literature, indicating the accuracy and consistency of the analysis.^44,45^

Fig. 2(d) illustrates the X-ray diffraction (XRD) pattern of calcium oxide nanoparticles coated with activated carbon. After subtracting the diffraction peaks of the electrophotocatalyst, AC shows diffraction peaks at 2*θ* degrees of 26.43°, which is assigned to the (002) (JCPDS 75-1621) *hkl* lattice parameter.^46^ CaO NPs show diffraction peaks at 2*θ* degrees of 32.22°, 37.37°, 53.71°, 64°, and 67.69°, which are assigned to the (111), (200), (202), (311), and (222) (JCPDS 82-1690) *hkl* lattice parameter reflections of the cubic CaO phase.^47,48^ The Debye–Scherrer formula (eqn (4)) was used to determine the crystallite size of the CaO NPs coated with AC, and the result was 27 nm.^49^ A void-free lattice structure is promoted by the decorations of cubic CaO and AC, and the surfaces get electrons from nearby atoms. The interface is increased, and the atomic gap is reduced due to the high occupancy of electrons on the surfaces of the NPs.7
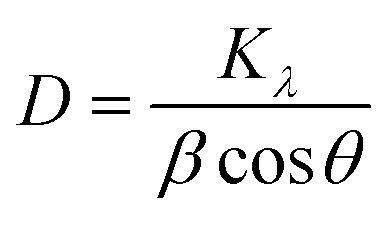
where ‘*D*’ is the crystalline size, ‘*K*’ represents the Scherrer constant (0.98), *λ* denotes the wavelength (1.54), and *β* denotes the full width at half maximum (FWHM).

To confirm the presence of carbon, oxygen, and calcium elements in the material, X-ray photoelectron spectroscopy (XPS) was performed. Ca 2p, O 1s, and C 1s are visible in the survey spectrum shown in Fig. 2(e). The C 1s peaks are exhibits in Fig. 2(f), where a binding energy of 284.5 eV shows the adsorbed C–C/C=C bonding groups and amorphous carbon.^50^Fig. 2(g) shows a more detailed spectrum of Ca 2p. The two separate peaks at 346 and 350 eV identify the part of CaO.^51,52^ The analysis of the O 1s spectra in Fig. 2(h) shows the binding energy with the deconvoluted peaks, which provides detailed information on the presence of (O_2_^−^), OH, and adsorbed CO_2_ in the CaO@AC nanocomposite. The XPS study explains the surface chemistry of the CaO@AC nanocomposite, which also offers essential information to maximize the performance of the NPs in different applications.

### 3.2. Structural analysis of CaO@AC

**Fig. 3:**
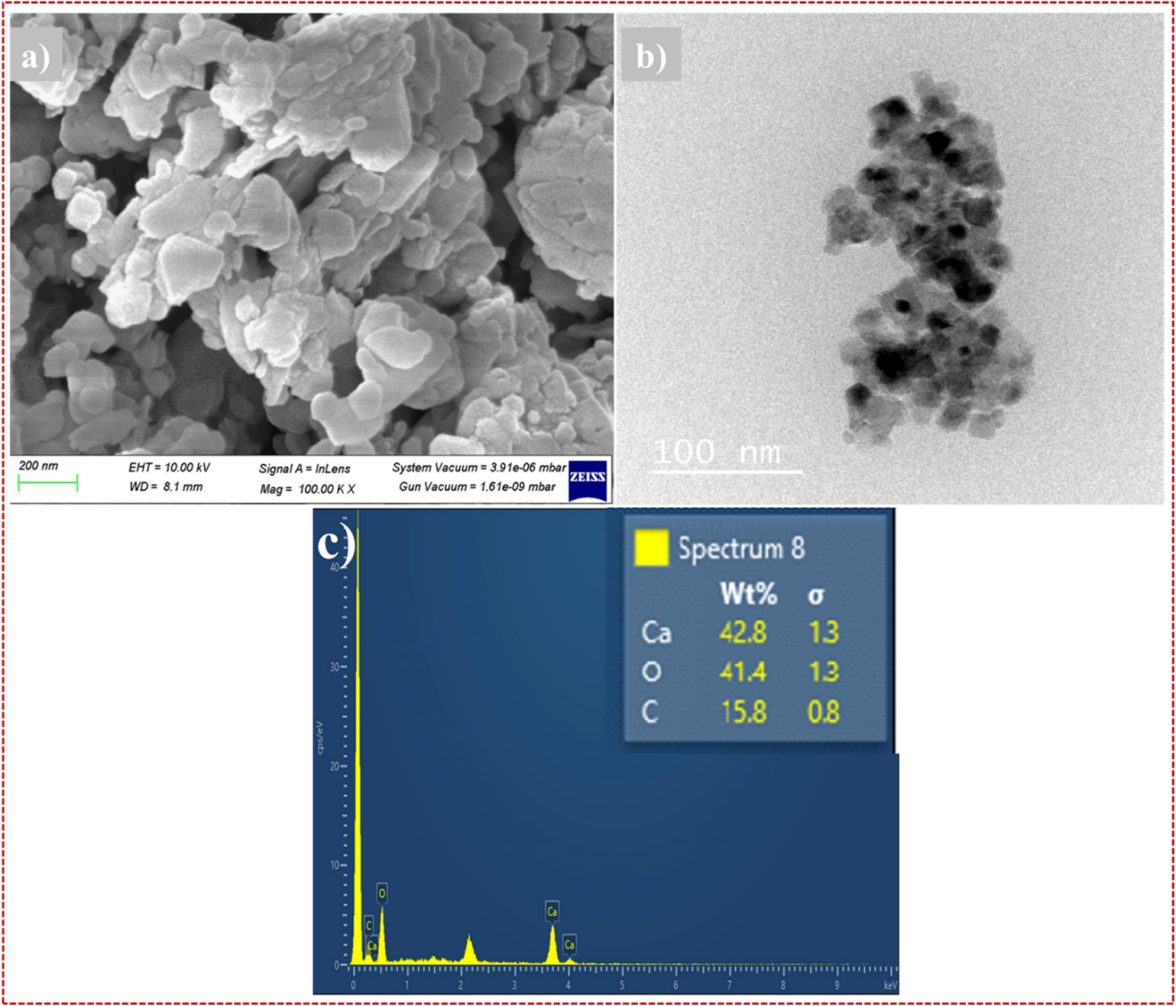
(a) SEM image, (b) TEM image, and (c) EDX spectra of CaO@AC. Experimental conditions: SEM analysis was performed at 30 kV using a Carl Zeiss microscope, and TEM analysis was conducted at 200 kV using an FEI Titan 80–300.

To understand the morphology of the obtained the CaO@AC nanocomposite, scanning electron microscopy (SEM) was performed (Fig. 3(a)). The bright spots shown in Fig. 3(a) indicate that CaO NPs were successfully incorporated inside the AC matrix. It is found that the doped CaO has a great effect on the morphology of AC NPs. Surface topography of CaO@AC can be seen in Fig. 3b, which shows an image captured by Transmission Electron Microscopy (TEM) that confirms the same morphology as the SEM pictures. For the dye molecules to stick to the NPs, they need to be grouped in a way that creates micropores, which can be seen in both the SEM and TEM images. The adsorption of electrolyte ions for energy storage, improvement of the degradation mechanism, and ease of dye molecule transfer are all made possible by these micropores. The TEM and SEM examinations show that the CaO NPs were successfully incorporated into the activated carbon matrix. The shape of material, including micropores and a high aggregation degree, suggests the nanoparticle potential for dye adsorption efficiency. In addition to the adsorption of dye molecules, this structure also promotes the transfer of dye molecule ions, which enhances the degradation of dye molecules and the ion storage mechanism of the dye.^53^

Material elemental composition analysis was also performed using energy-dispersive X-ray spectroscopy (EDS) (Fig. 3(c)). The EDX analysis shows that the material has C (0.27 keV),^54^ O (0.52 keV),^55^ and Ca (1.25 keV),^51^ respectively. The weight percentage of the NPs was further calculated by the EDS elemental composition analysis, which shows that the AC samples without impurities have a composition of 15.8% C, 41.4% O, and 42.8% Ca. Notably, a high calcium content was found in the nanocomposite.

## 4. Photocatalytic assays with CaO@AC

Photocatalytic activity was investigated through the degradation of a mixture of pollutants, which is known as a cocktail of pollutants–pollutants that are on the rise in wastewater. Since nanocomposites can interact with and degrade aqueous pollutants *via* various absorption methods, the degradation of the cocktail of pollutants provides a proof of concept. The catalytic activity of the synthesized catalysts for the degradation of model contaminants was evaluated under solar irradiation. Furthermore, the porous structure of the synthesized photocatalysts offered a greater number of water transport channels and exposed active sites, thereby improving their catalytic performance. To understand the degradation kinetics, the green-mediated synthesis of the CaO@AC nanocomposite was performed, and this composite was used to degrade different pollutants such as rhodamine B (RhB), rose bengal (RB), and methylene blue (MB). AC has a large surface area and a high adsorption capacity towards organic dyes; hence, a dark adsorption step was first carried out before irradiation with light. This procedure enabled stabilization of the adsorption equilibrium, and the initial concentration (*C*_0_), applied to calculate the photocatalytic reaction, was measured at the end of this equilibrium. As a result, the following reduction in the dye concentration in the presence of light can be explained primarily by photocatalytic degradation, but to a certain degree some adsorption contributes to the overall removal efficiency.

**Fig. 4:**
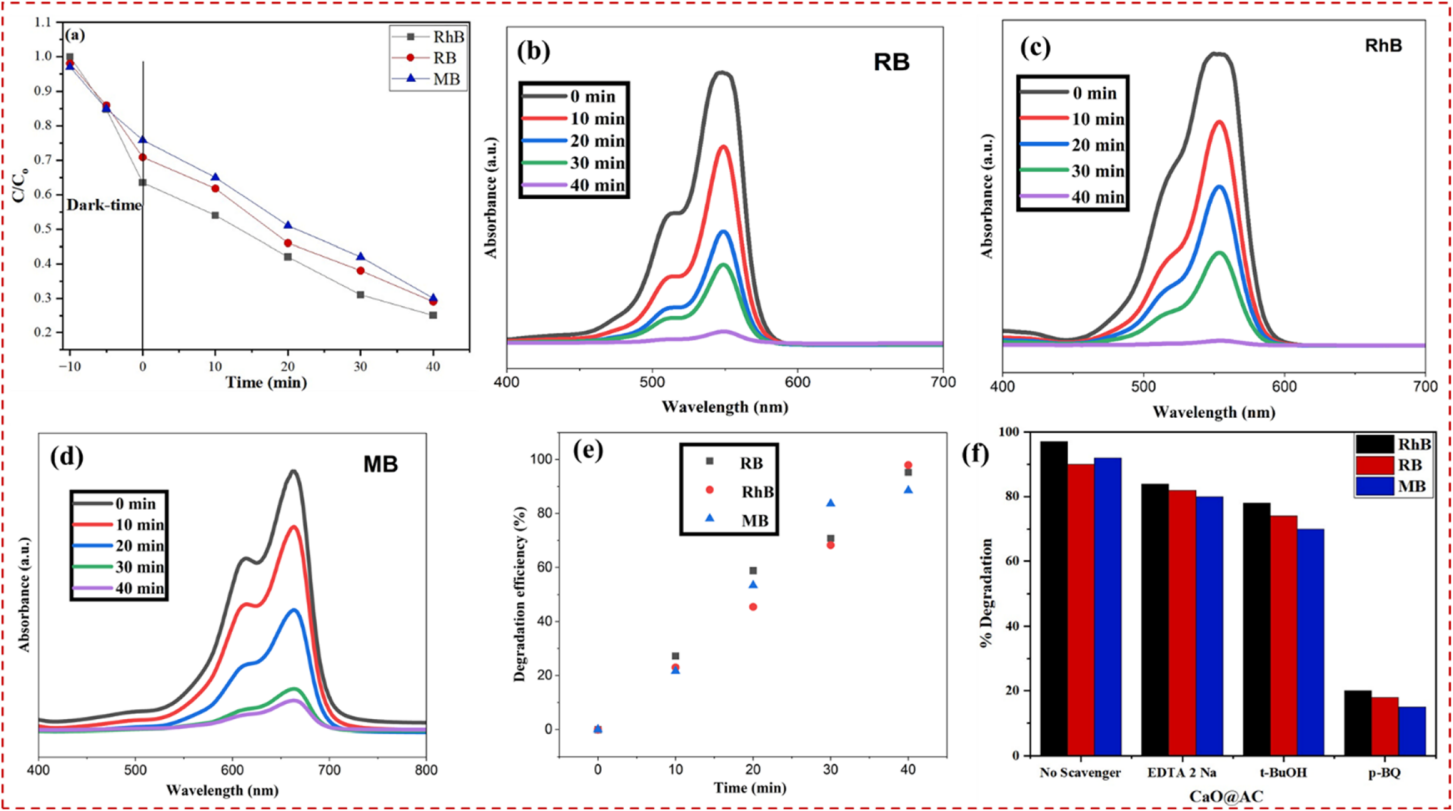
(a) Reaction mechanism under dark conditions without catalysts. (b–e) Variations in the degradation of different pollutants with time: (a) RhB, (b) RB, and (c) MB. (d) RB, MB, and RhB dye degradation efficiencies of CaO@AC. Experimental conditions: photocatalytic degradation was performed under simulated sunlight with 0.05 g of the CaO@AC catalyst in 50 mL of dye solution (1 × 10^−5^ M), stirring at 550 rpm. (f) Scavenger study for different pollutants.

The plot depicts the photocatalytic behavior of the degradation of RhB, RB, and MB dyes without concentrating on the dyes themselves by observing the normalized concentration (*C*/*C*_0_) with time. At first, in the dark stage (−10 to 0 min), all the dyes exhibit a minor decrease in *C*/*C*_0_, which is a consequence of the attachment of dye molecules onto the catalyst surface and establishment of adsorption–desorption equilibrium (Fig. 4(a)). When light irradiation (after 0 min) occurs, the constant decrease in *C*/*C*_0_ proves the beginning of the photocatalytic degradation. In these three dyes, RhB has the shortest degradation, and its lowest residual concentration is (∼0.25 at 40 min), compared to RB (∼0.29), MB (∼0.30) (see Fig. 4(a)), which is indicative of greater photocatalytic affinity and reactivity of RhB. The steeper slope of RhB indicates a higher generation rate and use of reactive species, while the less steep slope of MB indicates weak interaction with the catalyst surface or higher stability in the structure. Overall, the findings indicate a successful photocatalytic action of the material, where the efficiency of dye-based degradation is controlled by the adsorption behavior and molecular structure.

CaO@AC exhibits the highest degradation rate for RhB, which was recorded as 95% in just 40 min (Fig. 4(b)).^56,57^ Under solar irradiation, the degradation of pollutants increased to a maximum level compared to traditional reactions. After 40 minutes of exposure to sunlight, the strength of these absorption bands rapidly diminishes with illumination duration, and adsorption signals vanish almost entirely.^58^ On the other hand, the degradation performance of the CaO@AC nanocomposite was recorded at 86% and 74% for RB and MB, respectively (Fig. 4(c–e)). Fig. 4(d) shows that the degradation of different pollutants correlated with time. The primary absorption peaks were recorded at 554, 546, and 660 nm in the UV-vis spectra of RhB, RB, and MB, respectively.^59^ This rapid degradation was attributed to the synergistic coordination between AC and CaO NPs in the nanocomposite.

The bar chart shows effect of various scavengers on the photocatalytic degradation efficiency of RhB, RB, and MB dyes in the presence of CaO@AC catalyst, providing a picture of active reactive species that are engaged in the reaction (Fig. 4(f)). Without a scavenger, all three dyes have extremely high degradation rates (90–97%), which confirms high intrinsic photocatalytic activity of the material. However, when scavengers are added, there is a substantial reduction in the degradation efficiency. The degradation is moderately reduced with the introduction of EDTA-2Na (a hole scavenger) and *t*-BuOH (a hydroxyl radical scavenger) and is therefore determined by both photogenerated holes (h^+^) as well as hydroxyl radicals (˙OH) as the active species (Fig. 4(f)). Conversely, the efficiency of degradation decreases radically upon the addition of p-BQ (a superoxide radical, ˙O_2_^−^, and scavenger), which is a clear evidence that superoxide radicals contribute the greatest portion of the photocatalytic process. In general, it can be assumed that while various reactive species are engaged, the degradation process is mainly influenced by the ˙O_2_ radicals and h^+^ and ˙OH play secondary roles.

### 4.1. Mechanism for photocatalysis

**Fig. 5:**
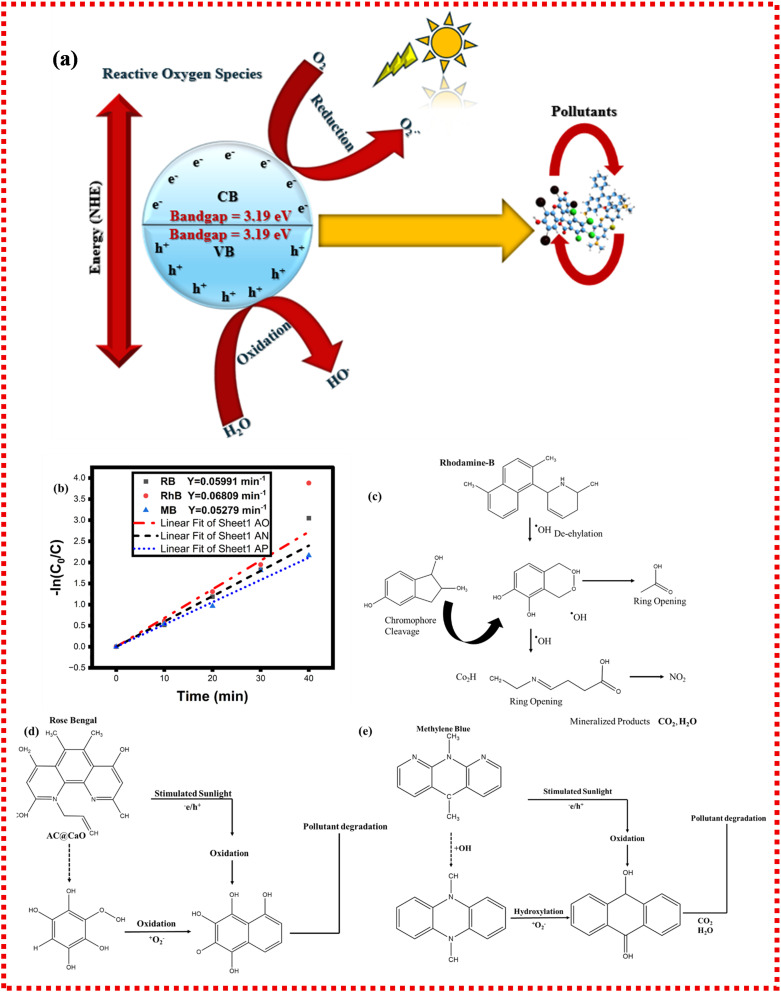
(a): Proposed mechanism of photocatalysis. (b) Shows the pseudo-first-order kinetic model of Rh–B: 0.06809 min^−1^, RB: 0.05991 min^−1^, and MB: 0.05279 min^−1^. (c–e) Schematic of the complete mineralization of Rh-b, RB, and MB into harmless products.

The principle behind this enhanced photocatalytic performance is essentially based on the semiconductor property of CaO and the adsorptive and conductive properties of AC.^60^ The approximate optical band gap of CaO@AC nanocomposite, from the analysis performed using UV-Vis spectroscopy, is about 3.19 eV. It is necessary to note that bulk CaO is generally found as a broad band-gap oxide in the literature. Thus, the value of the observed one is presumably relevant to the composite system, in which the interactions between CaO and activated carbon, as well as potential defects and interfacial states, can alter the optical absorption patterns (Fig. 5(a)). When the composite system is illuminated, photogenerated charge carriers can be produced, and the charge separation can be facilitated by the AC support, which is a kind of electron acceptor. These electrons can combine with the dissolved oxygen to form superoxide radicals (O_2_^−^), and holes help produce hydroxyl radicals (˙OH), and they can be used in the destruction of pollutants. The role of AC in this process is multi-faceted; as a high surface area adsorbent, it concentrates dye molecules around the active CaO sites and also accelerates degradation.^13^ Moreover, AC works as an electron reservoir and a conductive scaffold, which facilitates the separation of photoinduced charge carriers by decreasing electron–hole recombination.^61^ The separation of charges ensures a longer life of reactive species and the overall effectiveness of photocatalysis. The morphological examination using SEM and TEM validated the aggregated NP structure with microporous characteristics, thereby enhancing efficient mass transfer and accessibility of dye molecules to the catalyst surface.

Reaction pathways

8 AC@CaO + Energy → h^+^ + e^−^

9 H_2_O → H^+^ + OH^−^

10 h^+^ + OH^−^ → OH˙

11 e^−^ + O_2_ → O_2_˙^−^

12 O_2_˙^−^ + h^+^ → HOO˙

13 2HOO˙ → H_2_O_2_ + O_2_

14 H_2_O_2_ + UV → 2OH˙

15 2OH˙ + Model dyes → intermediates + minerals + CO_2_ + H_2_O

### 4.2. Pseudo-first-order kinetic model

The photocatalytic degradation performance of the produced CaO@AC nanocomposite towards three representative dyes, namely, RhB, MB, and RB, was methodically assessed under sunlight exposure. A pseudo-first-order kinetic model was employed to elucidate the kinetics of the dye degradation process. This approach is generally applicable when the starting dye concentration is minimal and catalyst dosage is excessive, hence simplifying the reaction rate expression to a first-order dependence on the dye concentration.

A pseudo-first-order kinetic model is expressed as follows:16
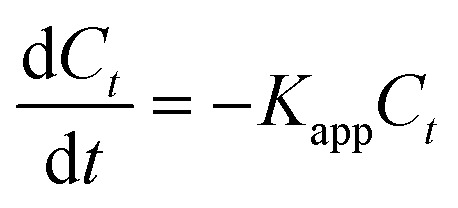
where: *C*_*t*_ = concentration of dye at time (*t*). *K*_app_ = apparent rate constant (min^−1^). *t* = time (min).

Integrated linear form:17
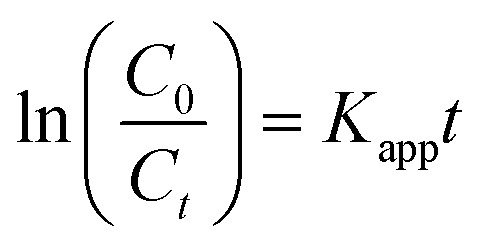


Or we can write as follows:

18 ln *C*_*t*_ = ln *C*_0_−*K*_app_*t*,

where *C*_0_ = initial dye concentration at *t* = 0.

Nonlinear form:

19 *C*_*t*_ = *C*_0_*e* − *K*_app_*t*

The linear plots against time for all three dyes are illustrated in Fig. 5(b) of the supplied image. The pronounced linearity (*R*^2^ = 0.99) substantiates that the degradation process adheres to the pseudo-first-order kinetics for all dyes. The thus determined rate constants (slopes of the lines) are as follows: RhB: 0.06809 min^−1^, RB: 0.05991 min^−1^, and MB: 0.05279 min^−1^. The results show that the fastest degradation is exhibited by Rh–B, followed by RB, and then MB.^62^ This order is consistent with that of the degradation efficiency as a function of energies displayed in Fig. 4(a). RhB has the highest degradation percentage (∼95%) within a time of 40 minutes, and thus is more susceptible to photocatalytic degradation under the given conditions. The observed kinetics have been attributed to several factors, *viz.*, efficient light absorption and electron–hole pair generation by CaO NPs, enhanced adsorption of dye molecules on the porous surface of AC, leading to a higher local dye concentration near the reactive sites, and synergistic interactions between AC and CaO inhibiting e^−^/h^+^ recombination to produce ROS of hydroxyl (˙OH) and superoxide (˙O_2_^−^) radicals to efficiently oxidize the dye molecules.^63^ As the pseudo-first-order correlation is high, the reaction rate is almost directly related to the availability of the molecules of dye and not limited by the mass transfer or other external effects.^66^ This result is in line with other studies on AC-supported metal oxides, where the adsorption–photocatalysis interface plays a key role in enhancing the reaction kinetics.^64^ This discovery corresponds with other research on AC-supported metal oxides, where the adsorption–photocatalysis interface is essential for improving reaction kinetics. The linear kinetic graphs and elevated correlation coefficients confirm that the photocatalytic degradation of RhB, MB, and RB dyes by the CaO@AC nanocomposite adheres to a pseudo-first-order kinetic model^36^ and is shown in Fig. 5(b). The determined rate constants further corroborate the enhanced photocatalytic efficacy of the compound, especially with RhB. These findings validate the material's capability as a swift and efficient photocatalyst for wastewater treatment purposes. Table 1 compares the photocatalytic efficiency of various photocatalytic materials over the CaO@AC nanocomposite.

**Table 1:** Comparison of the photocatalytic efficiency of various photocatalytic materials with the CaO@AC nanocomposite

Sr. No.	Photocatalytic material	Light source	Dosage	Degradation efficiency	Time	Ref.
1	TiO_2_/C	Visible light	10 mg	100% RhB	60 min	65
2	Co–NiF/AC	16 W low-pressure mercury lamps	100 mg	91% RhB	60 min	66
3	WO_3_/AC	400 W power metal halide lamp	10 mg	76% RhB	180 min	67
4	AC/Ag	80 W lamp	60 mg	96.09% MB	100 min	45
5	AC/CaO	—	500	MB	350 min	68
6	CaO/AC/ZnO	100 W incandescent light bulb	50 mg	96.7% MB	120 min	69
7	AC/CaO/Fe_3_O_4_	—	200	Formaldehyde	150	70
**8**	**CaO@AC**	**Sunlight**	**0.05 grams**	**95%, 74%, and 86% for RhB, MB, and RB**	**40 min**	**This work**

## 5. Antimicrobial activity of the CaO@AC nanocomposite

**Fig. 6:**
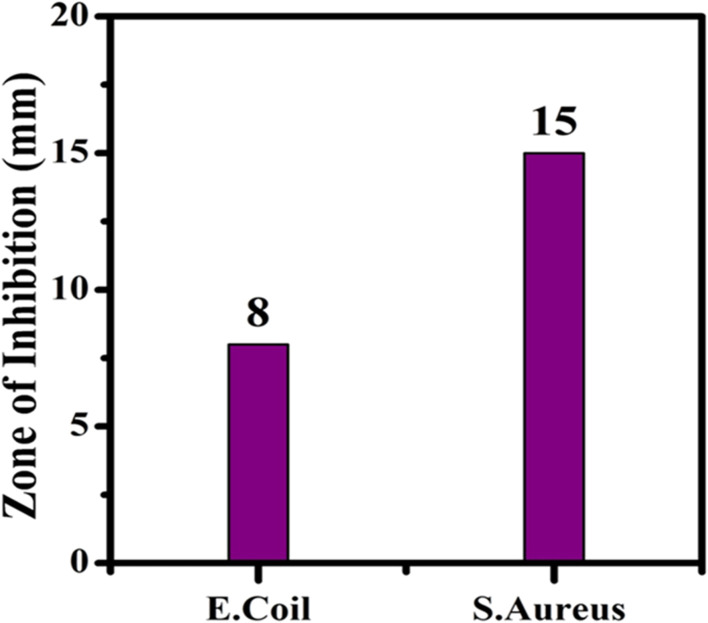
Zone of inhibition of the CaO@AC nanocomposite.

Using the agar well-diffusion technique, the antibacterial properties of the nanocomposite were evaluated against strains of Gram-positive *S. aureus* and Gram-negative *E. coli* bacteria. In the antimicrobial experiment used to assess the antagonistic activity of the CaO@AC nanocomposite, the zone of inhibition generated by the common medication streptomycin (20 mm) was compared. According to the findings, an 8 mm zone of inhibition for *E. coli* and a 15 mm zone for *S. aureus* are shown in Fig. 6. These results suggest that Gram-positive and Gram-negative bacteria are significantly toxic to the CaO@AC nanocomposite. The observed toxic effect against both types of bacteria suggests the potential of the CaO@AC nanocomposite as an effective antibacterial agent.

## 6. Electrochemical performance of bifunctional photoelectrocatalysts

**Fig. 7:**
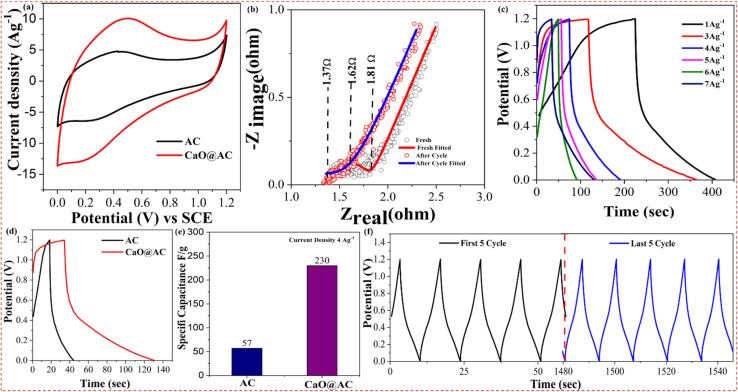
(a) CV analysis of AC and CaO@AC. Experimental conditions: CV analysis was conducted using an electrochemical workstation at a 100 mV s^−1^ scan rate in a 3 M KOH electrolyte. (b) Nyquist plot analysis of CaO@AC before and after cycling. Experimental conditions: EIS measurements were conducted in a 3 M KOH electrolyte with an amplitude of 10 mV in the frequency range of 0.01 Hz to 100 kHz. (c) GCD analysis of AC and CaO@AC at 1 to 7 A g^−1^ (d) GCD analysis of AC and CaO@AC at 4 A g^−1^ (e) GCD specific capacitance of AC and CaO@AC at 4 A g^−1^. (f) Long-term cyclic stability of CaO@AC. Experimental conditions: GCD measurements were performed at 4 A g^−1^ current density in a 3 M KOH electrolyte.

Cyclic voltammetry (CV) is a valuable method for elucidating electrode materials' capacitive and energy storage applications. In a 3 M KOH aqueous electrolyte, the CV curves of the three electrodes shown in Fig. 7(a) exhibit a rectangular and symmetrical shape for the AC electrode with a scan rate of 100 mV s^−1^. The capacitive behavior of CaO@AC in the voltage range of 0 to 1.2 V closely resembles the ideal capacitive behavior of AC,^71^ even with a pseudo-capacitance contribution from CaO particles.^72^ In contrast, the CaO@AC nanocomposite electrode displays an enlarged CV area compared to the AC electrode, indicating enhanced electron adsorption capacity due to the surface functionalities of CaO on the AC matrix. This enhancement is attributed to the combination of the large surface area's double-layer capacitance of AC and the redox-type reaction characteristics of CaO. This result can be ascribed to the increased specific surface area of the CaO@AC nanocomposite electrode and its abundance of electrochemically active sites, particularly CaO. These factors facilitate increased capacitance through a pseudocapacitive contribution enabled by the electrode's intense contact with the electrolyte. In addition, the CV curve analysis was performed through preliminary experiments, and the potential range was selected from 0 to 1.2 V because it was recommended to have stable capacitive behaviour without any distortion in the CV curves. As the potential range increased from 1.2 V, the electrolyte showed decomposition effects and caused side reactions.

Electrochemical impedance spectroscopy (EIS) was employed to investigate the impact of CaO doping on the charge storage capacity. The EIS electrodes were performed in a 3 M KOH electrolyte. The Nyquist plots for both the AC and CaO@AC samples are presented in Fig. 7(b). The impedance plots exhibit a semicircle in the high-frequency region and a linear component in the low-frequency zone, demonstrating nearly comparable characteristics. For an optimal design of an electrode material for an electric double-layer capacitor (EDLC), typically a linear component with a slope approaching 90° with respect to the imaginary axis *Z* is exhibited. The high-frequency region, where the intersection of *Z* real axis, includes the region of the interface between the current collector and CaO@AC sample. As shown in Fig. 7(b), the CaO@AC sample, post-cycling, shows a smaller semicircle and a slight deviation from the straight line on the *Z* imaginary axis as compared with the CaO@AC sample without cycling. These features suggest good capacitive performance and low-charge-transfer resistance across the interface. The charge-transfer resistance (*R*_ct_) of the CaO@AC sample noticeably decreases from 1.81 Ω before charge–discharge (CD) cycling to 1.62 Ω after the cycling, which indicates enhanced binding, good stability and increased surface activation of the electrode.^73^ The decrease in the semicircle value also further supports the enhanced electrochemical performance of the CaO@AC sample and hints at the decreased impedance at the electrode–electrolyte interface.^74^ Accordingly, the galvanostatic charge–discharge spectra with 1 to 7 Ag^−1^ of current density showed coulombic efficiency that indicates that the nanocomposite has excellent capacitive behaviour and also indicated electrochemical reversibility (Fig. 7(c)). The GCD analysis of the AC and CaO@AC nanocomposite electrode was systematically evaluated at a current density of 4 A g^−1^, as shown in Fig. 7(d). The absence of a charge–discharge curve is an indication of an intense electrical contact and a well-established electrode/electrolyte interface. The observed charge–discharge behavior also confirmed the electrochemical properties of CaO@AC, which were in agreement with the CV curves. Using GCD at a given current density, the specific AC and CaO@AC capacitances were measured as 57 F g^−1^ and 230 F g^−1^, respectively (Fig. 7(e)). A very important factor is the cycle life of a supercapacitor electrode. The capacitance retention analysis revealed its amazing stability and long-term performance, demonstrating that CaO@AC retained more than 99% of its capacitance after 5000 cycles (Fig. 7(f)). The enhanced surface functionality of the CaO@AC is responsible for its enhanced reactivity at higher power densities. The increased surface functionality of CaO@AC explains its improved reactivity at higher power densities. These functional groups are hypothesized to promote faster responses and better performance at higher power densities by facilitating quick ion channels, ion mobility, and charge-transfer activities inside the pseudo-capacitance. The CaO@AC nanocomposite presented in this study exhibits a specific capacitance of 230 F g^−1^ at 4 A g^−1^ in 3 M KOH, with an outstanding 99% capacitance retention after 5000 cycles. However, a complete device design was not constructed in this work, and the electrochemical properties were analyzed by using a three-electrode system, which shows that the nanocomposite has good rate capability, high capacitance, and cycling stability. Based on these outputs, future work will be focused on designing a symmetric/asymmetric system by using this nanocomposite. Table 2 presents a comparison of the electrochemical efficiency of various electrode materials over the CaO@AC nanocomposite.

**Table 2:** Comparison of the electrochemical efficiency of various electrode materials with the CaO@AC nanocomposite

Sr. No.	Electrode material	Specific capacitance	Electrolyte	Capacity retention	Ref.
1	CuO/MPC	616 F g^−1^ 1 A g^−1^	2 M KOH	69% at 5000 cycles	75
2	MnO_2_/N–AC	424 F g^−1^ 1 1 A g^−1^	6 M KOH	85.4% at 10 000 cycles	76
3	Bi_2_O_3_–GO NC	933 F g^−1^ 14 A g^−1^	6 M KOH	80% at 5000 cycles	77
4	AC/NiO	624.20 F g^−1^ at A g^−1^	6 M KOH	98% at 1000 cycles	78
5	BiFeO_3_-NC	811 F g^−1^	1 M Na_2_SO_4_	98% at 1000 cycles	79
6	NiCo_2_O_4_@NC	2000 F g^−1^ at 1 A g^−1^	6 M KOH	79.8% at 5000 cycles	73
7	**CaO@AC**	**230 F g^−^** ^**1**^ **at 4 A g^−^** ^**1**^	**3 M KOH**	**99% at 5000 cycles**	**This work**

## 7. Ragone plot

**Fig. 8:**
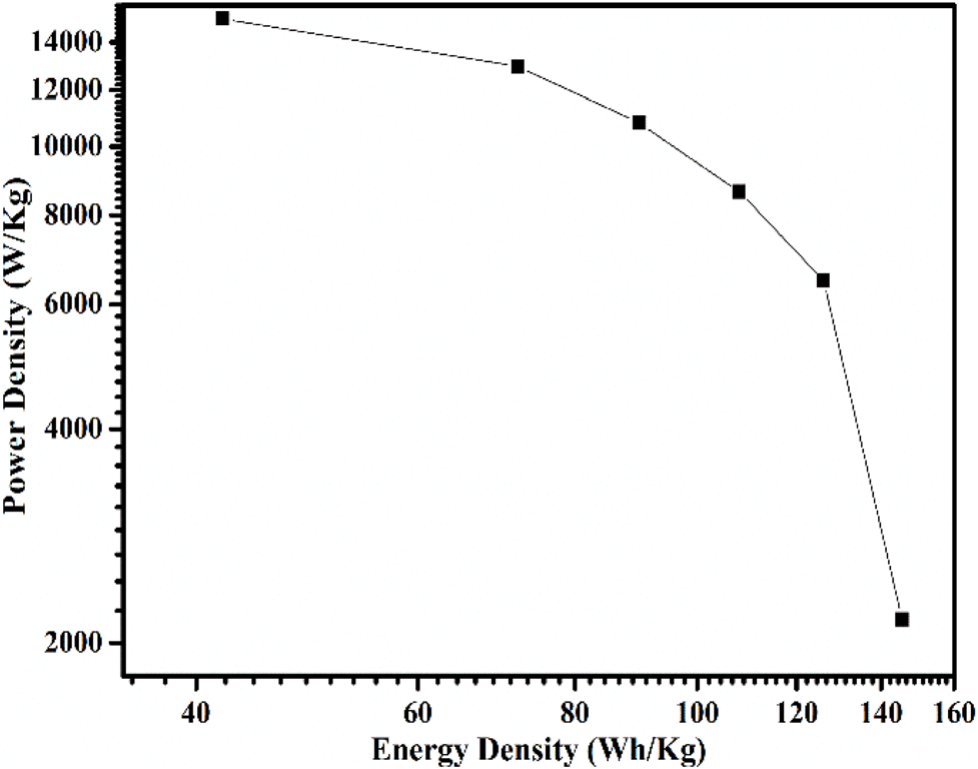
Ragone plot of the CaO@AC nanocomposite.

The Ragone plot of the CaO@AC nanocomposite electrode shows that there is a good balance between its power density and energy density, which would make it a high-performance supercapacitor electrode (Fig. 8). At a lower power density (about 60–70 W kg^−1^), the electrode provides a high energy density of about 15 000–16 000 W kg^−1^. The energy density decreases gradually with the power density, reaching a point of approximately 6000–7000 W kg^−1^ at −120 W kg^−1^ and even less at −140 to −150 W kg^−1^. This is common with supercapacitive systems because the diffusion of ions is limited and the internal resistance is high at high charge–discharge rates. However, the comparative low rate of decrease in power density with energy density suggests that the CaO@AC electrode has excellent rate capability. This can be explained by the fact that the electric double-layer capacitance of activated carbon and pseudocapacitive contribution of CaO nanoparticles are synergistic, hence the improved performance. Activated carbon has a porous structure which facilitates the quick movement of ions and the collection of charges, and CaO endows more redox-active sites, which increases the total capacity of charge storage greatly. Moreover, the lower charge-transfer resistance at EIS analysis is conducive to efficient delivery of energy at large power outputs. These results verify that the CaO@AC nanocomposite is a viable solution to the high energy density to high power density ratio, which is why it is one of the front runners in the innovation of electrochemical energy storage applications.

## 8. Conclusion and recommendations

The structural, morphological, photocatalytic, and antibacterial properties of the CaO@AC nanocomposite synthesized using a green synthesis technique are thoroughly investigated in this work. The homogenous integration of CaO@AC moieties is corroborated by analysis of the results of XRD, FTIR, EDX, and SEM. The photocatalytic experiments conducted using simulated solar irradiation show that the CaO@AC nanocomposite degrades dyes efficiently, achieving 95%, 74%, and 86% degradation efficiencies for RhB, RB, and MB, respectively, in 40 minutes, and offers good recycling capacity. Furthermore, investigating the antibacterial activity demonstrates that the CaO@AC nanocomposite is very toxic to both *S. aureus* and *E. coli*, severely inhibiting their growth. This photocatalyst offers a viable method for eliminating environmental contaminants. Moreover, we calculated specific capacitances of 57 F g^−1^ for AC and 230 F g^−1^ for the CaO@AC nanocomposite using the galvanostatic charge–discharge analysis at a certain current density. Remarkably, the induced current from the CaO@AC nanocomposite exceeded that of raw activated carbon, indicating its greater capacitance. Different features such as large surface area, double-layer capacitance, and the redox-type capacitor properties are responsible for the improved performance, indicating that CaO@AC nanocomposite are a potential option for supercapacitor applications. The practical application of the CaO@AC nanocomposite faces some limitations and challenges, which should be addressed to make them ideal candidates for broad spectrum applications. These are as follows:

✓ The SEM analysis shows that the aggregated structure affects their broad-spectrum applications and limits their photocatalytic applications.

✓ Although the CaO@AC nanocomposite shows promising degradation efficiency, their recyclability test and long-term stability should be studied under harsh environmental conditions to make them ideal candidates.

✓ The formation of the reactive species during photocatalysis may result in the formation of undesired by-products. To combat this, the removal of the targeted contaminants without detrimental end products is a challenging task.

✓ Green synthesis of nanomaterials needs a long-time heat transformation reaction. Therefore, their scale-up synthesis approach could be difficult and will not be cost-efficient.

✓ Nanomaterials also show antimicrobial characteristics, and hence, their potential environmental impact should be evaluated comprehensively.

✓ The synergistic effect of nanocomposites should be evaluated for the large-scale applications of clean energy production.

✓ Challenges related to the cathode material deformation should be overcome by introducing advanced defect engineering approaches.

✓ The synergistic effect of nanomaterials in handling wastewater that holds intricate mixes of contaminants requires more examination.

✓ The effect of reaction conditions on NPs' properties should be further evaluated.

✓ The potential recovery and reusability of the nanomaterials without any loss should be investigated for sustainable development.

Addressing these challenges through targeted research will help to analyze the full potential of the CaO@AC nanocomposite in photocatalysis and energy storage applications.

## Conflicts of interest

The authors declare that they have no known competing financial interests or personal relationships that could have appeared to influence the work reported in this paper.

## Data Availability

Data will be provided upon reasonable request to authors.
